# Supplementary material for: Network isolators inhibit failure spreading in complex networks
Source: Nat Commun. 2021 May 25;12:3143. doi: 10.1038/s41467-021-23292-9 (PMC8149673; doi:10.1038/s41467-021-23292-9)
Supplement: Supplementary file 1 — Supplementary Information [file 41467_2021_23292_MOESM1_ESM.pdf]

Supplementary Information for
Network isolators inhibit failure spreading in complex networks

Franz Kaiser,[1,2] Vito Latora,[3,4,5,6] and Dirk Witthaut[1,2]

[1]*Forschungszentrum Jülich, Institute for Energy and Climate Research (IEK-STE), 52428 Jülich, Germany*
[2]*Institute for Theoretical Physics, University of Cologne, Köln, 50937, Germany*
[3]*School of Mathematical Sciences, Queen Mary University of London, London E1 4NS, UK*
[4]*Dipartimento di Fisica ed Astronomia, Università di Catania and INFN, 95123 Catania, Italy*
[5]*The Alan Turing Institute, The British Library, London NW1 2DB, UK*
[6]*Complexity Science Hub Vienna, 1080 Vienna, Austria*

(Dated: April 8, 2021)

This Supplementary Material contains four Supplementary Notes and twelve Supplementary Figures.

Supplementary Figures 3

Supplementary Notes 15
Supplementary Note 1: Flow networks 15
Supplementary Note 2: Description of link failures 19
Supplementary Note 3: Network isolators inhibit failure spreading completely 20
Supplementary Note 4: Linear controllability of complex networks 23

Supplementary References 24

SUPPLEMENTARY FIGURES

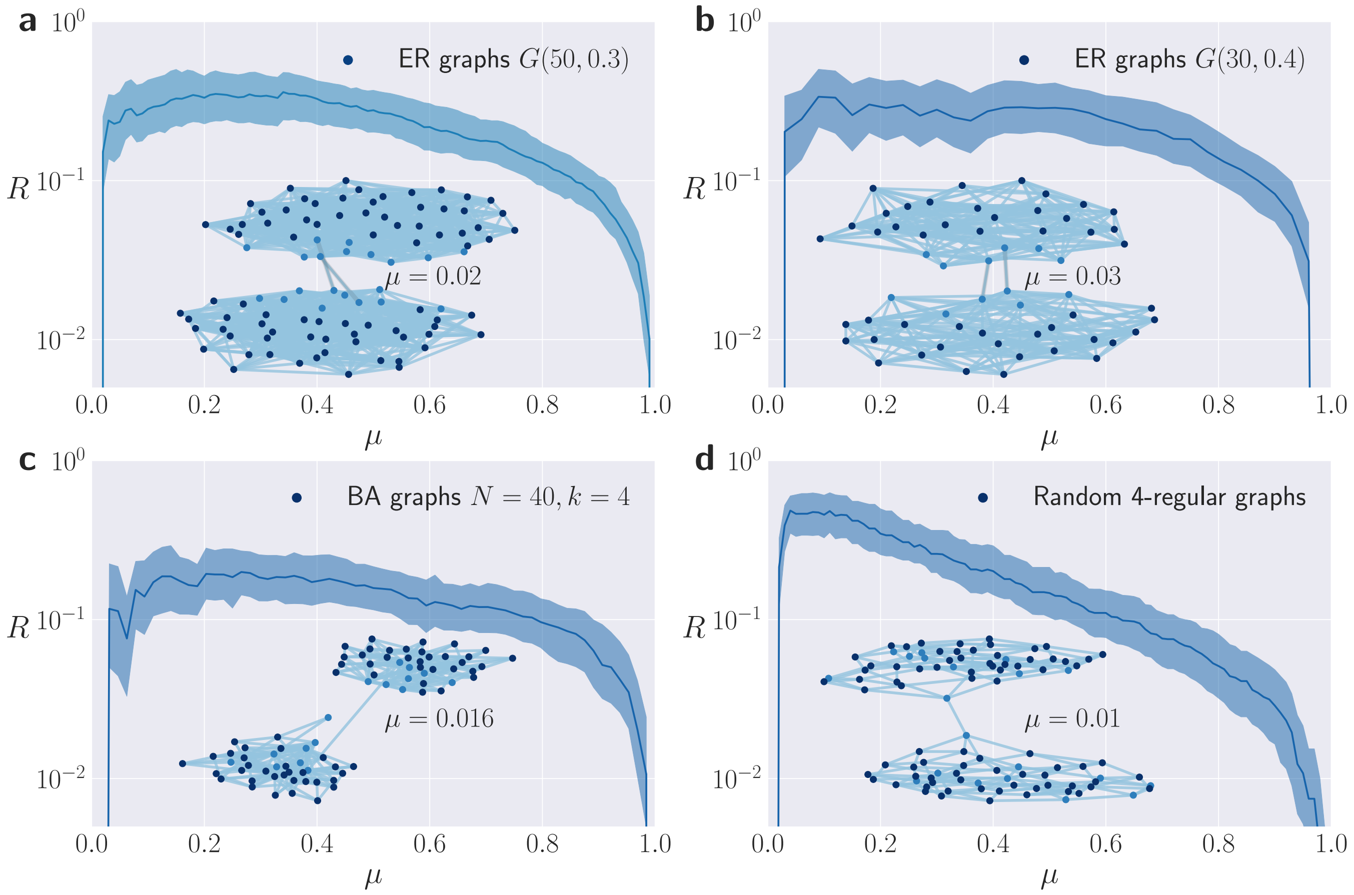

Supplementary Figure 1. **Averaged ratio of flow changes decays with high and low connectivity for different random graphs.** All panels show ratio of flow changes R averaged over all links and distances against connectivity parameter μ (see methods) along with corresponding graph for low values of the connectivity parameter. **a** Two ER graphs with parameters $N_i = 50, p_i = 0.3$ connected with probability $\mu = 0.02$ at a randomly chosen share of $c = 0.2$ their nodes. **b** Same as in (a), but with parameters $N_i = 30, p_i = 0.4, \mu = 0.03, c = 0.2$. **c** A similar scaling is observed if two BA random graphs with parameters $N_i = 40, k_i = 4$ are connected with probability $\mu = 0.016$ at a randomly chosen share of $c = 0.2$ their nodes. **d** The scaling is also preserved if two 4-regular, random graphs are connected with parameters $N = 50, \mu = 0.01, c = 0.2$. Blue line represents median value over all distances and shaded region indicates 0.25- and 0.75-quantiles for all graphs.

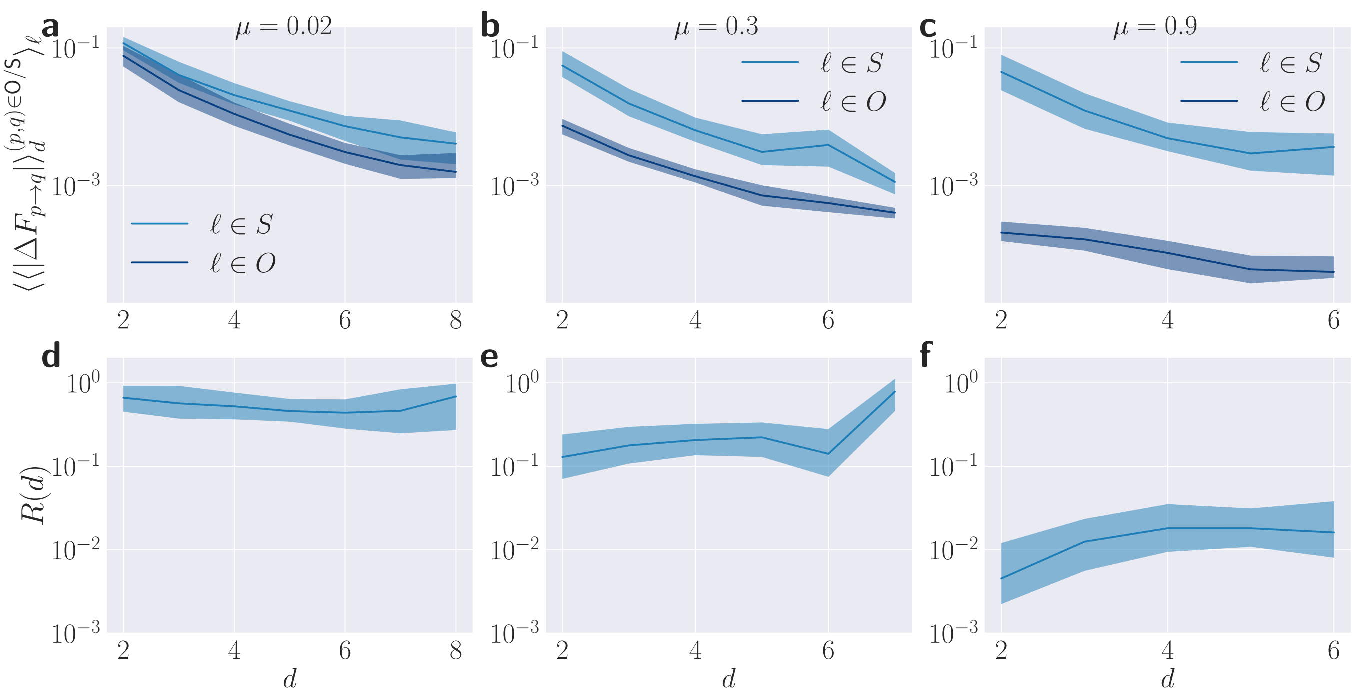

Supplementary Figure 2. **Ratio of flow changes depends weakly on distance.** We examine the scaling of link flow changes with distance for two ER random graphs $G(120, 0.02)$ that are connected at $c = 0.2$ nodes with changing probabilities $\mu = 0.02$ (left), $\mu = 0.3$ (centre) and $\mu = 0.9$ (right). We only consider the largest component from each of the two random graphs and remove all dead ends as they result in vanishing flow changes. **a** to **c** Normalised absolute flow changes decay with distance when averaging over all possible trigger links. We always assume a unit flow on the failing link before the failure. We distinguish flow changes in the same (blue, top) and the other (purple, bottom) module of the graph. Flow changes are consistently higher in the same module for all distances. **d** to **f** Ratio of flow changes averaged over all possible trigger links $R(d)$ reveals a weak dependence of the ratio on distance. Blue line represents median value over all distances and shaded region indicates 0.25- and 0.75-quantiles for all graphs.

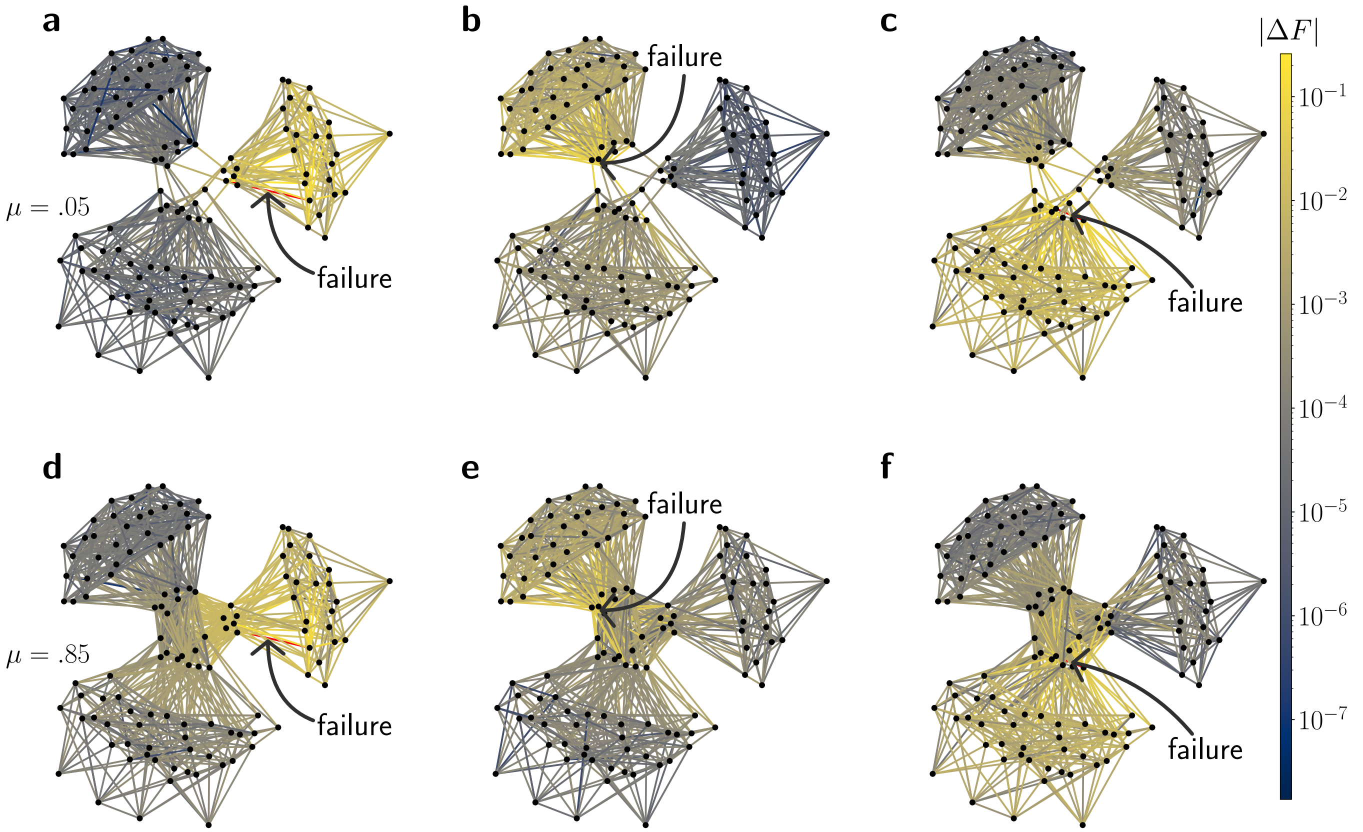

Supplementary Figure 3. **Increasing or decreasing connectivity between more than two modules reduces failure spreading equally well.** Here, we demonstrate a possible extension of the synthetic network model described in the Methods section to more than two modules. For each panel, we simulate a single link failure (red) that results in flow changes (colour coded). **a** to **c** Three ER random graphs $G(30, 0.3)$ (right), $G(50, 0.2)$ (bottom) and $G(40, 0.4)$ (top left) that are mutually interconnected with probability $\mu = 0.05$ at 20 percent, i.e., $c = 0.2$, thus resulting in three mutually weakly connected modules. **d** to **f** Connecting the same modules as shown in a to c with probability $\mu = 0.85$, thus resulting in strong inter-module connectivity, reduces failure spreading equally well.

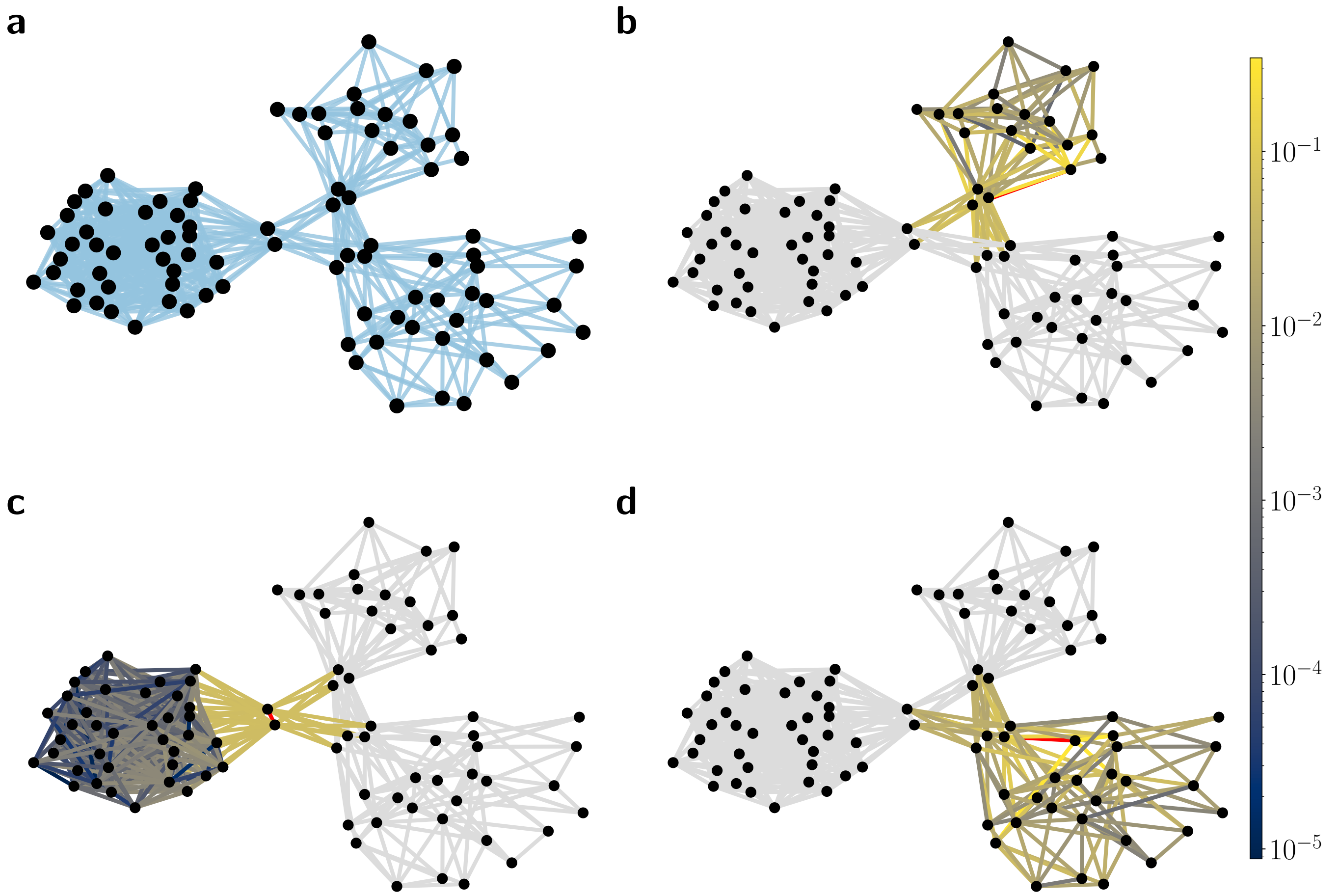

Supplementary Figure 4. **Networks isolators can be generalised to network consisting of more than two modules. a** Topology of a network consisting of three ER random graphs $G(40, 0.4)$ (left), $G(20, 0.3)$ (top) and $G(30, 0.2)$ (bottom right) that are mutually connected through network isolators. **b** to **d** Link failures in each of the individual subgraphs (red lines) do not change flows (colour code) in any of the other subgraphs.

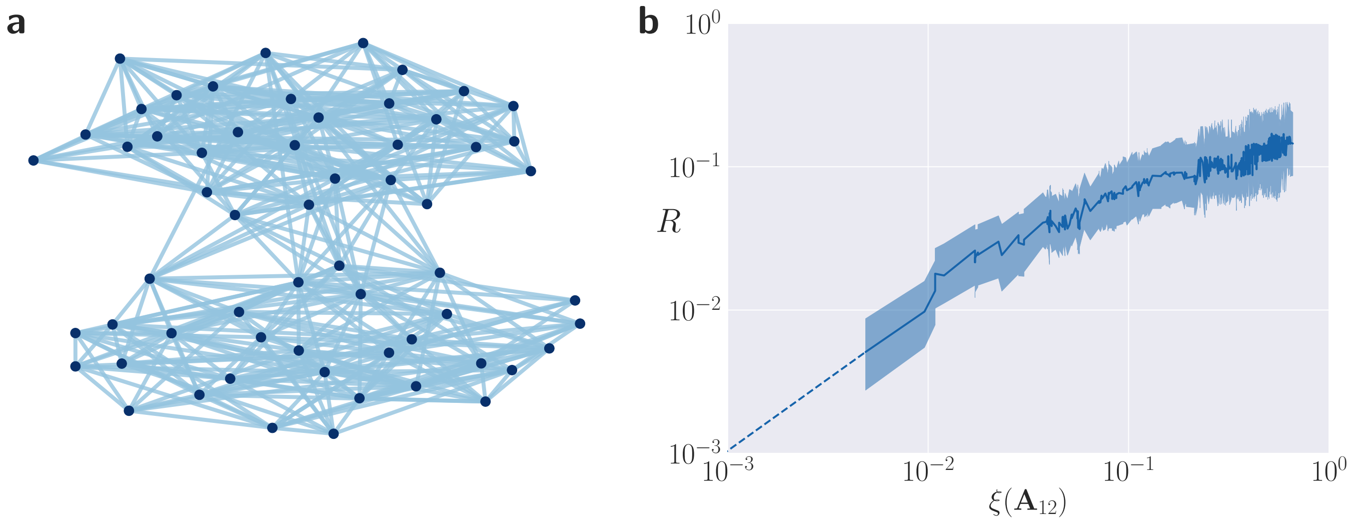

Supplementary Figure 5. **Robustness of network isolators shows the same scaling with perturbations for different graphs.** Robustness of network isolators measured by ratio of flow changes R averaged over all links against measure of perturbations to network isolators $\xi(\mathbf{A}_{1,2})$. **a** Graph created from the graph ensemble and shown in Fig. 1c was modified in such a way that it contains a network isolator connecting five nodes from one part to five nodes of the other part through a bipartite connectivity structure. Edge weights are drawn randomly from a normal distribution $\mathcal{N}(10, 1)$ except for the network isolator where the randomly chosen weights of five edges starting in the same node and connecting to all connecting nodes in the other part were chosen as basis weights for all other connections between the two parts. **b** The isolator robustness shows qualitatively the same scaling as for the 6-regular graph shown in Fig. 1c. Perturbations were applied in 1000 repetitions choosing a perturbation strength of $\alpha = 0.05$. Dotted line takes into account the fact that the curve goes through the point $\xi = R = 0$ for a perfect isolator.

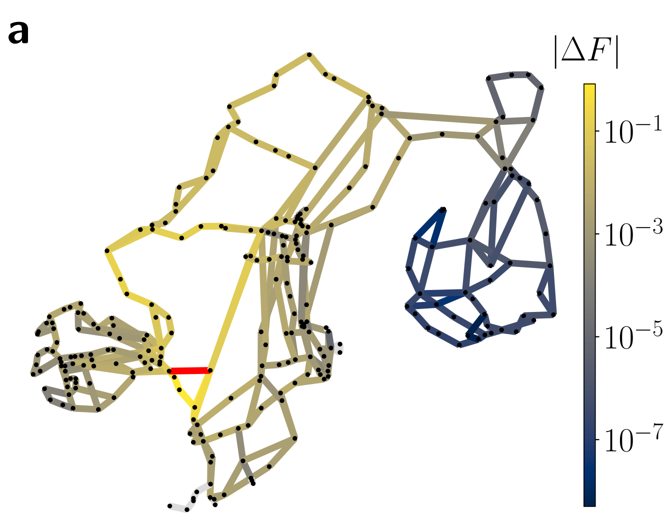

Supplementary Figure 6. **Network isolators do not generally increase grid vulnerability.** **a** Failure of a link with unit flow in the Scandinavian grid before the construction of the network isolator yields a strong response in terms of absolute flow changes $|\Delta F|$. **b** After adding two links to create a network isolator (blue shaded region, see Figure 3c), we simulate a failure of one of the links *in* the isolator. We observe that both, the failure within the isolator (panel b) as well as a failure in the initial grid in close proximity to the location where the isolator is constructed (panel a) yield a similar effect. In this case, the network's vulnerability is thus not increased by including the network isolator. However, a failure in the isolator may potentially affect the whole network.

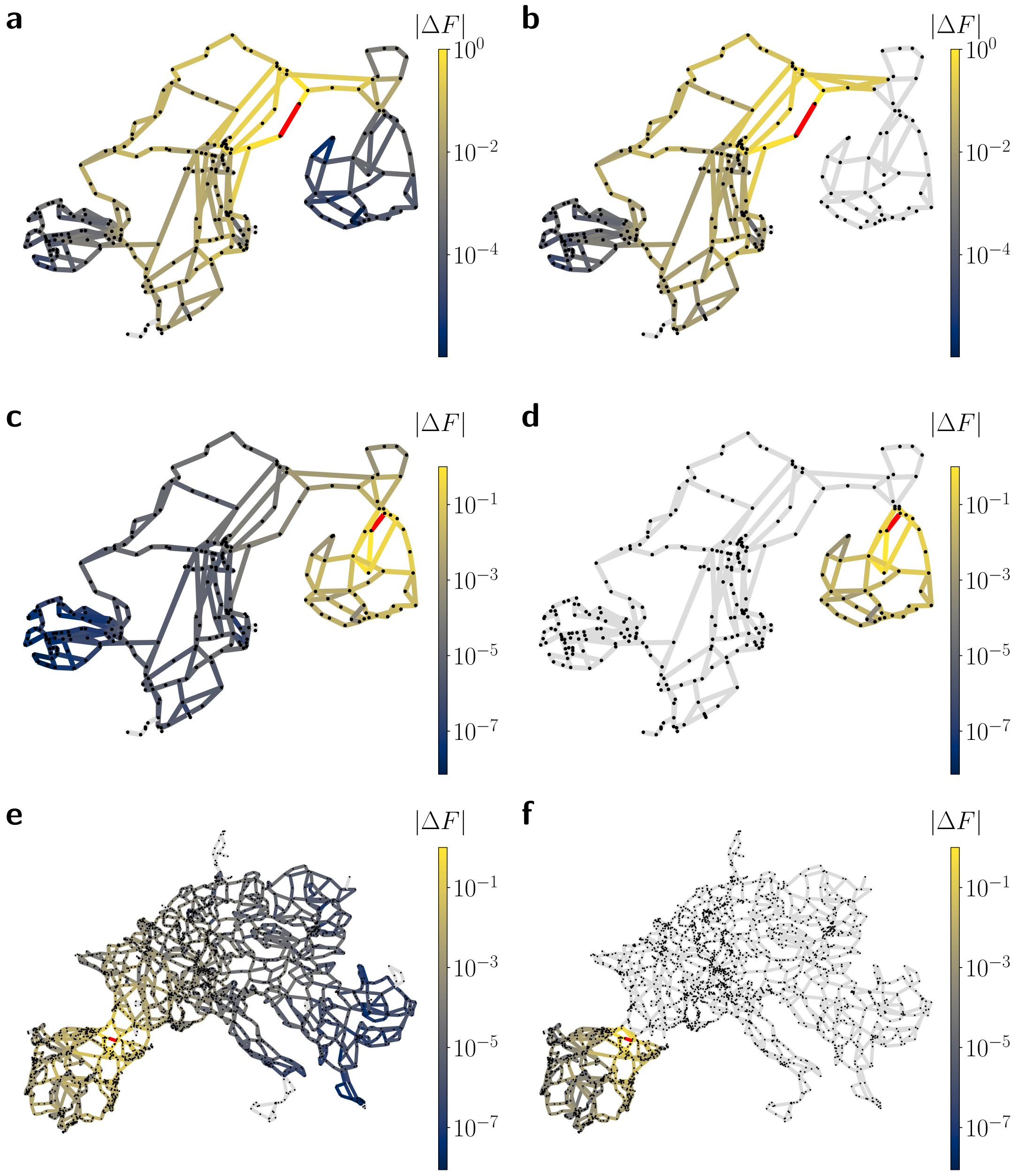

Supplementary Figure 7. **Network isolators may be realised in various real-world power grids.** All grid topologies and line susceptances were extracted from the open European energy system model PyPSA-Eur, which is fully available online[1]. **a,c,e** Initial failure of a link (red) with unit flow results in flow changes in the whole network for Scandinavia a,c as well as the central European grid e. **b,d,f** After introducing network isolators to the grids, failure spreading to other parts of the network is completely stopped. The construction of isolators follows the "recipes" illustrated in Figure 3.

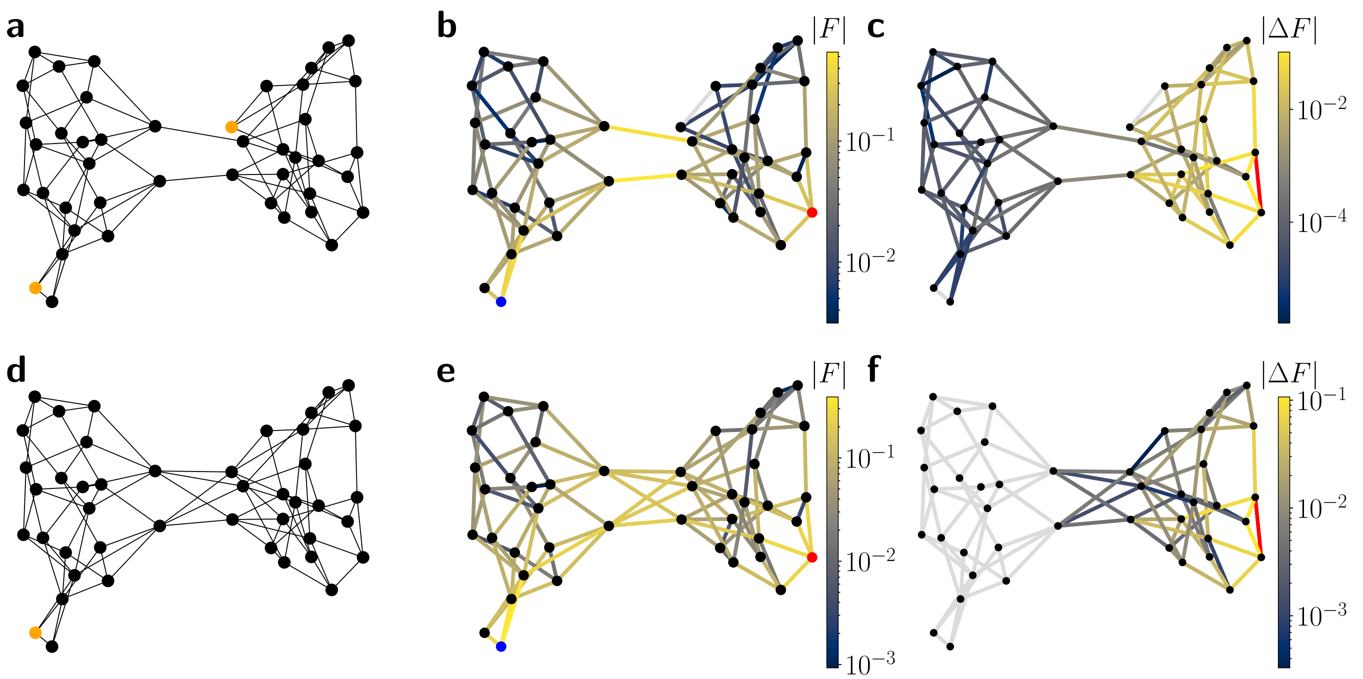

Supplementary Figure 8. **Isolators do not generally prevent the controllability of a network.** **a** An example of an undirected network with two weakly connected components that requires $N_D = 2$ driving nodes (in orange) to be controlled. This can be calculated from the graph adjacency matrix, which has, by construction, an eigenvalue $\lambda^M = -1$ with algebraic multiplicity $\delta(\lambda^M) = 2$ (See Eq. 20 and Ref. [2]). **d** After adding a few links to create a network isolator, we have $N_D = 1$ and only one node (colored orange) is necessary to control the entire network, i.e., the network isolator has in this case increased the controllability of the network. **b** We show the flows obtained by our linear flow model for a single source of power $P = 1$ at the node colored in red and a single sink with $P = -1$ at the node colored in blue. The resulting (absolute) flows are color-coded: The flow can easily reach from the red node to the blue node. **e** Adding the isolator, flow can still propagate freely from the source node (red) to the target node (blue) in the same way as in panel b. Hence, the isolator does not prevent the propagation of flows. **c** Simulating the failure of a single link (red), we observe that flows do also change in the other part of the network. **f** Conversely, the isolator does prevent propagation of flow changes caused by a link failure in the right part of the network to its left part.

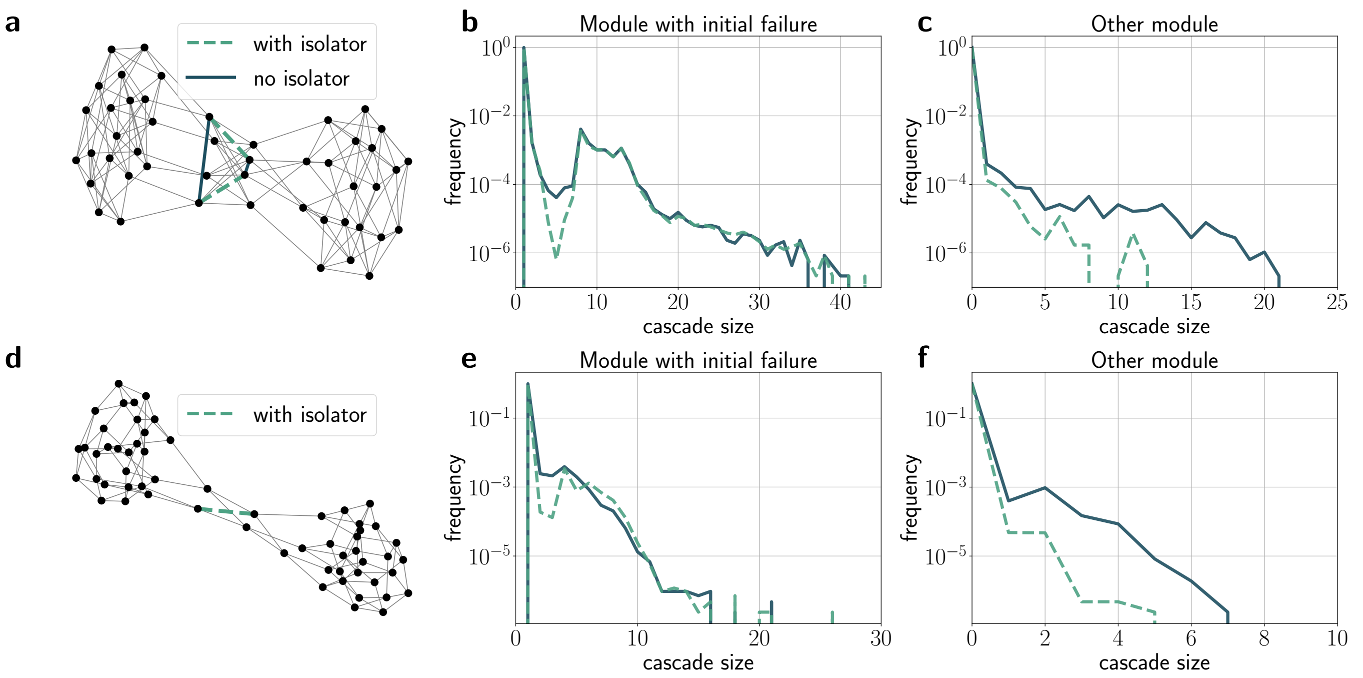

Supplementary Figure 9. **Cascade propagation is strongly suppressed in the presence of network isolators.** **a** We consider the six-regular graph shown in Figure 1c with unit edge weights and $7 \cdot 10^4$ different initial conditions where we randomly assign 25% of the nodes to be sources with $P_i = 2$ and the remaining ones to be sinks with $P_i = -\frac{2}{3}$. We then simulate the failure of any possible link in the left module of the network for each initial condition using the linear flow model and monitor the size of the resulting cascade of failures, setting the line limit to $F_{i\to j}^{\max} = 1.0$ (see Methods). We compare two different graphs: the six-regular graph containing a network isolator (light green, dotted) and a corresponding six-regular graph where to links have been rewired (dark green). **b,c** For both graphs, we compare the cascade sizes in the module where the failure was triggered (b) and the other module (c). As a result, cascade sizes are significantly smaller if the other module is shielded by a network isolator although the overall connectivity between the modules is higher in this case. **d-f** We perform the same set of simulations for the graph shown in panel d which conforms the result of reduced cacade sizes in the presence of network isolators. Parameters for panels d-f are given by $P_i = 0.9$ for sources and $P_i = -0.3$ for sinks.

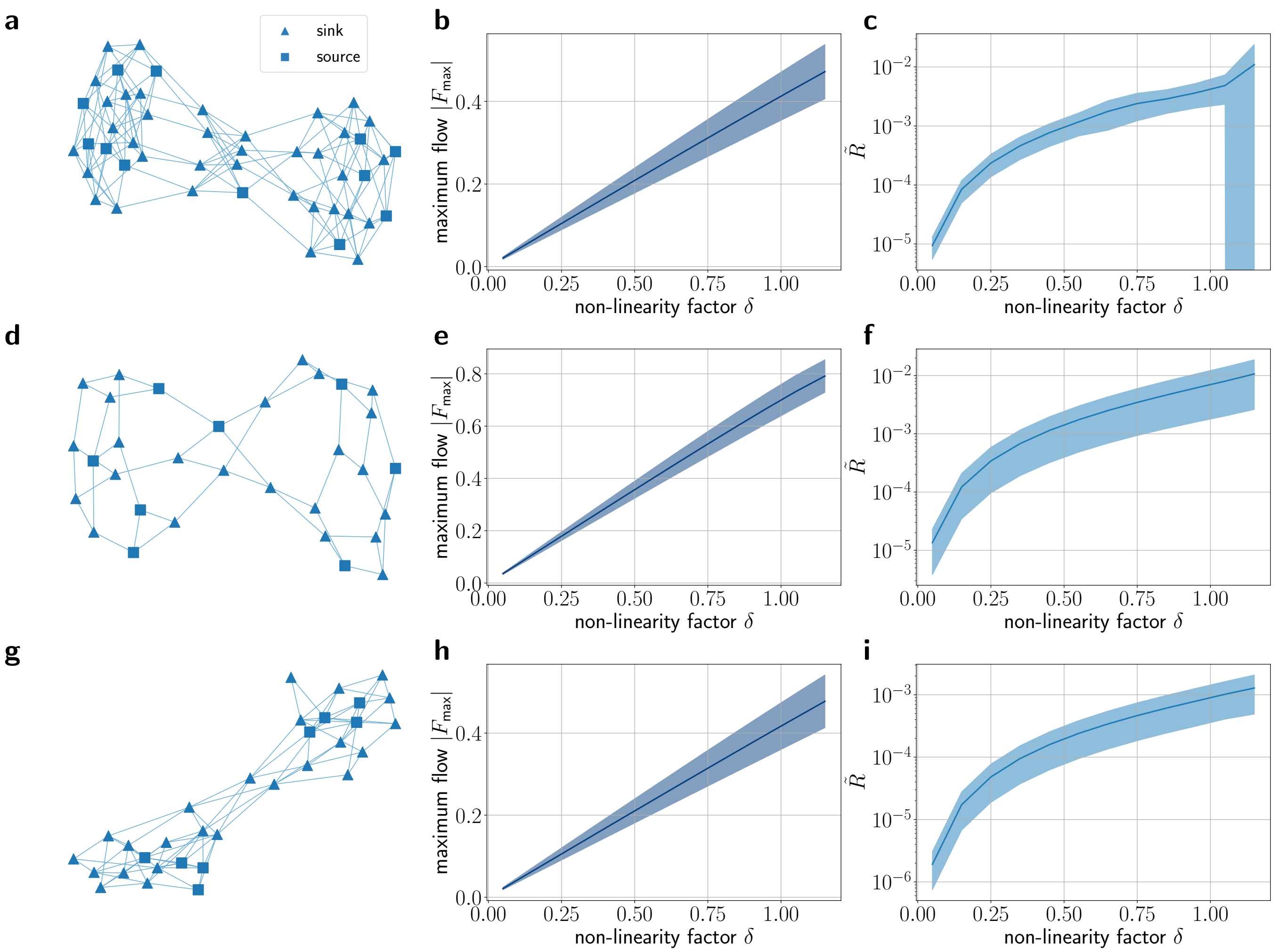

Supplementary Figure 10. **Network isolation effect persists for non-linear flows.** **a** We consider the six-regular graph shown in Figure 1c and simulate 50 different initial conditions where we randomly assign 25% of the nodes to be sources with $P_i = 0.9 \cdot \delta$ and the sinks correspondingly to balance the sources. Here, δ is a prefactor tuning the degree of non-linearity in the non-linear flows $\tilde{F}_{i\to j} = A_{i\to j} \cdot \sin(\vartheta_i - \vartheta_j)$. **b** For each initial condition, we analyse the maximum flow in the network $|F_{\max}|$ as an indicator of non-linearity for different degrees of non-linearity δ. **c** We then evaluate the ratio $\tilde{R}$ of non-linear flow changes which is obtained from Eq.(8) by replacing the flow changes ΔF by their non-linear counterpart and averaging over all distances and trigger links in the left module. To examine to what extent network isolators prevent perturbation spreading from the left module to the right module, we plot this ratio against the non-linearity factor. With increasing degree of non-linearity, there is no longer exact isolation, i.e. $R = 0$, but a strong shielding effect persists. **d-i** We perform the same type of analysis for two three-regular graphs (d) and two random graphs $G(16, 0.3)$ (g) connected via network isolators and observe a similar scaling of the ratio $\tilde{R}$ with the non-linearity factor. Shaded regions indicate half a standard deviation evaluated over all initial conditions for all plots.

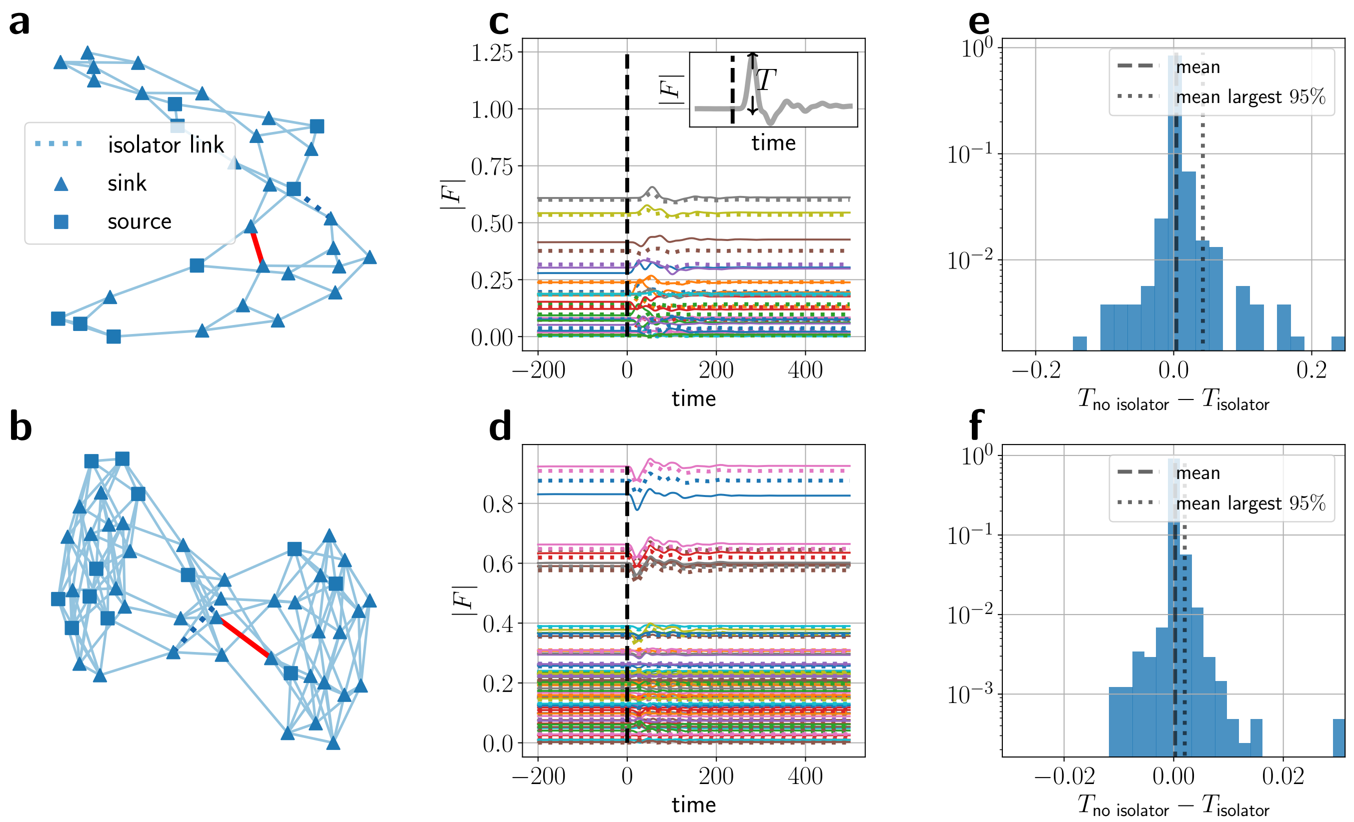

Supplementary Figure 11. **Transient amplitudes are slightly reduced in the presence of network isolators. a** We analyse a network consisting of two modules that are connected via three links and add a fourth link (dotted) to create a network isolator. We randomly assign 25% of the nodes to be generator nodes (squares) and the remaining ones to be load nodes (triangle). We then simulate the removal of a single link (red) and monitor the corresponding response in the dynamic nonlinear system described by the second order Kuramoto model (Eq. (10)). **c** Non-linear dynamics of the flows in the upper module after the failure of a single link at time zero (dotted, vertical line) in the network before (straight lines) and after the addition of the isolator link (dotted lines). We monitor the maximum Amplitude T of the transient dynamics comparing the fixed point before and after the failure (inset). **e** To analyse the impact of network isolators on transient overloads, we compare the transient amplitudes before ($T_{\text{no isolator}}$) and after (T_{isolator}) constructing the isolator in the upper module for all possible link failures in the lower module. In most cases, the transient amplitudes stay the same after introducing the network isolator as confired by the mean close to zero (black, dashed line). However, evaluating only the 95% changes in amplitudes with the largest changes in magnitudes (dotted line), we observe a significant shift towards positive values indicating a reduced risk of transient overloads when network isolators are present. **b,d,f** The result is confirmed by performing the same analysis for a different network containing a larger network isolator. Inertia constants are given by $M = 1$ and damping constants by $D = 0.3$ for all nodes and panels (see Eq. (10)).

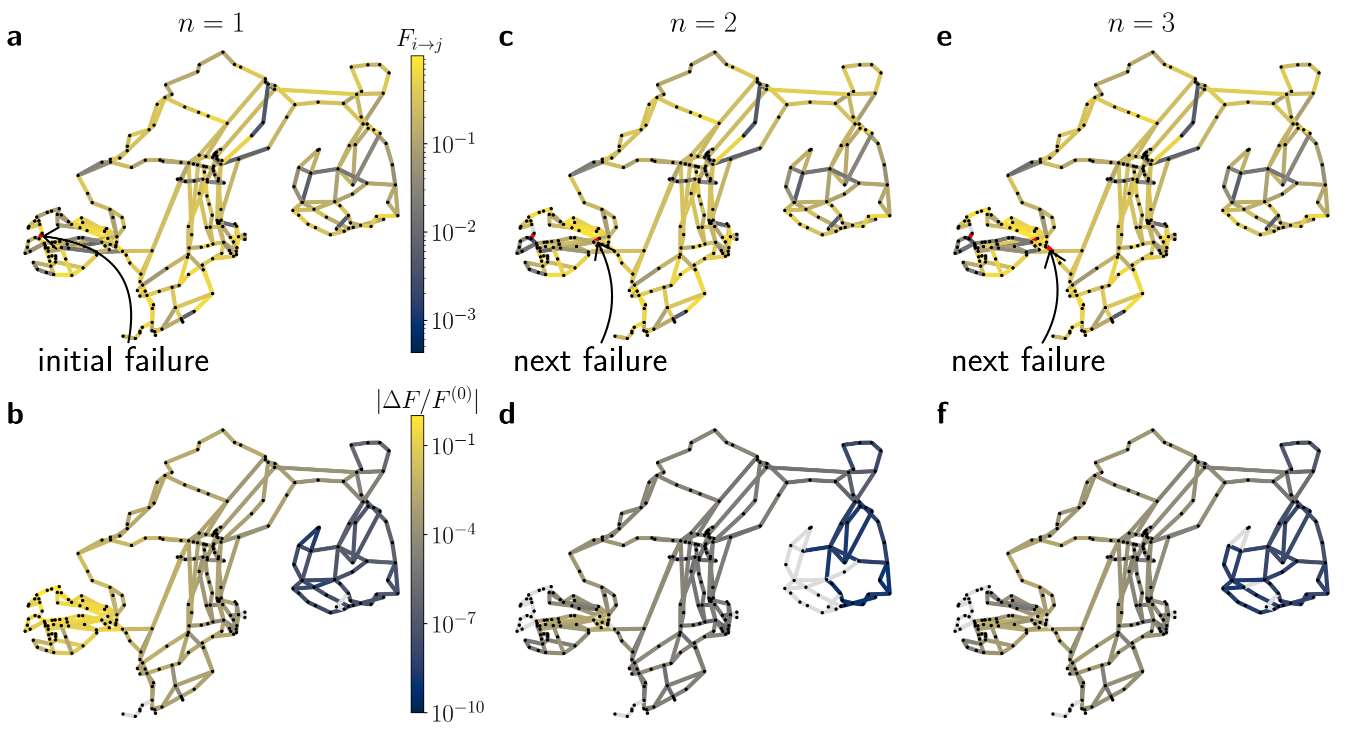

Supplementary Figure 12. **Non-locality of cascade propagation and decay of flow changes** We illustrate the first three steps of the cascade in the Scandinavian power grid for shown in Figure 4 in the main text for the grid without a network isolator. **a** Line loading in the Scandinavian grid prior to the initial failure with the initially failing link highlighted. Note that line loading is heterogeneously distributed within the network. **b** Relative flow changes $|\Delta F_\ell / F_{\text{fail}}^{(0)}|$ for any link ℓ as a result of the failure of the link shown in (a). Flow changes are normalized by the flow carried by the failing link $F_{\text{fail}}^{(0)}$ (a, arrow) before the failure, such that the maximum relative flow change is unity. The flow changes clearly decay with distance from the failing link. **c** Line loading after the initial failure: although flow changes decay with distance, the next failing link is relatively far apart from the initially failing link when considering the geographic or geodesic network distance. **d** Relative flow changes after the failure of the link shown in c, normalised again by the flow that the link shown in c carries before the failure. Again, the flow changes are localised. **e** Line loading after the failure of both links shown in panels a and c. The next failure is closer to the failing link shown in c, but even farther apart from the initially failing link, leading to an overall non-local cascade of failures. **f** Again, relative flow changes as a result of the link failure shown in panel e are strongly localised.

SUPPLEMENTARY NOTES

Supplementary Note 1: Flow networks

In this section, we briefly review the theory and applications of linear flow networks.

Mathematical description

In this work, the main model of interest is a linear flow network model which we introduce more formally in this section. Consider a connected graph $G = (E, V)$ consisting of $N = |V|$ nodes and $L = |E|$ edges. Assign to each node in the network a potential $\vartheta_n \in \mathbb{R},\ n \in V(G)$ and to each edge a weight $A_{ij} \in \mathbb{R}^+,\ \ell = (i,j) \in E(G)$. Now we assign a *flow* $F_{i\to j} \in \mathbb{R}$ to each link $\ell = (i,j) \in E(G)$ in the network that is assumed to be linear in the potential drop

$$F_{i\to j} = A_{ij} \cdot (\vartheta_i - \vartheta_j) = -F_{j\to i}. \tag{1}$$

Suppose that there are sources and sinks attached to the nodes of the networks $P_i \in \mathbb{R},\ i \in V(G)$. In this case, the in- and outflows at each node have to balance with the sources and sinks

$$P_i = \sum_{k=1}^{N} F_{i\to k}. \tag{2}$$

This equation is known as continuity equation or *Kirchhoff's current law.* If the sources and sinks P_i are given, Eqs. (2) and (1) completely determine the potentials in the network (up to a constant shift to all potentials). In a power grid, the sources and sinks are the power injections or withdrawals as a result of power production or consumption, respectively. When looking at the stable, operational fixed point of a power grid they are balanced such that $\sum_i P_i = 0$ – we therefore assume this to hold in the following sections. The theory of linear flow networks applies resistor networks, as well as AC power grids in the DC approximation, hydraulic networks and networks of limit cycle oscillators, which will be discussed in detail in this section.

Now we introduce a compact, vectorial notation which facilitates the analysis of perturbations or damages to the network. Note that the flow is a signed quantity that depends on the orientation of the edges that we arbitrarily fix for this purpose and say that the flow is directed from node i to node j in this case. We can write the flows in vectorial notation $\vec{F} = (F_1, ..., F_L)^\top \in \mathbb{R}^L$ as follows;

$$\vec{F} = \boldsymbol{K}\boldsymbol{I}^\top \vec{\vartheta}. \tag{3}$$

Here, $\boldsymbol{K} = \mathrm{diag}(K_1, ..., K_L) \in \mathbb{R}^{L\times L}$ is the graph's weight matrix that collects the edge weights and $\boldsymbol{I}^\top$ is the transpose of the the graph's edge-node incidence matrix $\boldsymbol{I} \in \mathbb{Z}^{N\times L}$ that determines the orientation of the graph's edges by the following relationship

$$I_{j\ell} = \begin{cases} +1 & \text{if edge } \ell \,\hat{=}\, (j,k) \text{ starts at node } j, \\ -1 & \text{if edge } \ell \,\hat{=}\, (j,k) \text{ ends at node } j\ , \\ \ \ 0 & \text{otherwise.} \end{cases} \tag{4}$$

Furthermore, $\vec{\vartheta} = (\vartheta_1, ..., \vartheta_N)^\top \in \mathbb{R}^N$ is a vector of potentials or voltage phase angles. We can also define a vector of power injections $\vec{P} = (P_1, ..., P_N)^\top \in \mathbb{R}^N$ such that the continuity equation reads as

$$\vec{P} = \boldsymbol{I}\vec{F}. \tag{5}$$

In this expression, the correspondence between the power balance and *Kirchhoff's current law* becomes manifest: it states that the in- and outflows at each node have to balance the injections and withdrawals of power. Combining Eq.s (3) and (5), we may find a relationship between angles $\vec{\vartheta}$ and power injections $\vec{P}$, thus defining the graph's weighted *Laplacian matrix* $\boldsymbol{L} = \boldsymbol{I}\boldsymbol{K}\boldsymbol{I}^\top \in \mathbb{R}^{N\times N}$, by

$$\vec{P} = \boldsymbol{I}\boldsymbol{K}\boldsymbol{I}^\top \vec{\vartheta} = \boldsymbol{L}\vec{\vartheta}. \tag{6}$$

The weighted Laplacian matrix used here has the following entries

$$L_{ij} = \begin{cases} -A_\ell & \text{if } i \text{ is connected to } j \text{ via } \ell = (i,j), \\ \sum_{\ell=(i,k)\in E(G)} A_\ell & \text{if } i = j, \\ 0 & \text{otherwise.} \end{cases} \tag{7}$$

The Laplacian matrix plays an important role in graph theory [3]. If the underlying graph G is connected, it has one zero eigenvalue $\lambda_1 = 0$ with corresponding eigenvector $\vec{v}_1 = \vec{1}/\sqrt{N}$. Therefore, the matrix is not invertible. In many cases, it would nevertheless be desirable to invert the matrix, e.g. in order to find the phase variables given the power injections in Eq. (6). This problem is typically overcome by making use of the matrix's *Moore-Penrose pseudoinverse* $\boldsymbol{L}^\dagger$. It may be used to invert Eq. (6) in the same way as for the ordinary matrix inverse in the case of balanced power injections [4]. The Moore-Penrose pseudoinverse of the graph Laplacian $\boldsymbol{L}$ allows for the following representation: using $\boldsymbol{L}$'s eigenvalues sorted by magnitude $\lambda_1 = 0, \lambda_2 \leq ... \leq \lambda_N$ with corresponding eigenvectors $\vec{v}_1 = \vec{1}/\sqrt{N}, \vec{v}_2, ..., \vec{v}_N$, we can express its pseudoinverse $\boldsymbol{L}^\dagger$ as [5]

$$\boldsymbol{L}^\dagger = (\vec{v}_1, \vec{v}_2, ..., \vec{v}_N) \begin{pmatrix} 0 & 0 & ... & 0 \\ 0 & \lambda_2^{-1} & ... & 0 \\ ... & ... & ... & ... \\ ... & ... & ... & \lambda_N^{-1} \end{pmatrix} (\vec{v}_1, \vec{v}_2, ..., \vec{v}_N)^\top .$$

The second eigenvalue λ_2 is usually referred to as *Fiedler eigenvalue* or *algebraic connectivity* and is an indicator of the graph's overall connectivity. If we assume the overall graph to be connected, this eigenvalue is strictly greater than zero $\lambda_2 > 0$. Importantly, a large difference between second and third eigenvalue $\lambda_3 - \lambda_2$ implies a strong modularity in the graph and thus indicates the existence of a community structure [6–8].

Before we proceed, let us briefly fix the notation for the following sections: we will refer to an edge $\ell = (\ell_1, \ell_2) \in E(G)$ and its index ℓ in the ordered set of all edges interchangeably or refer to it by its terminal nodes ℓ_1 and ℓ_2. If we assume the edge space to be spanned by vectors in the two element field $GF(2)$, we may express the edge by a unit vector $\vec{l}_\ell = (0, ..., \underbrace{1}_{l}, ..0)^\top \in GF(2)^L$ which we refer to as the edge's indicator vector. The edge-node incidence matrix $\boldsymbol{I}$ then maps this unit vector to the corresponding unit vectors in the field of vertices $GF(2)^N$. We thus get the following result for the edge expressed in terms of its starting vertex ℓ_1 and terminal vertex ℓ_2:

$$\vec{\nu}_\ell = \boldsymbol{I} \cdot \vec{l}_\ell = \vec{e}_{\ell_1} - \vec{e}_{\ell_2} = \begin{pmatrix} 0 \\ ... \\ 1 \\ ... \\ -1 \\ ... \end{pmatrix} \begin{matrix} \\ \\ \}\ell_1 \\ \\ \}\ell_2 \\ \\ \end{matrix},$$

where $\vec{e}_{\ell_1}$ and $\vec{e}_{\ell_2}$ are basis vectors in $GF(2)^N$

$$\vec{e}_{\ell_1} = \begin{pmatrix} 0 \\ ... \\ 1 \\ ... \\ ... \\ 0 \end{pmatrix} \}\ell_1, \quad \vec{e}_{\ell_2} = \begin{pmatrix} 0 \\ ... \\ ... \\ 1 \\ ... \\ 0 \end{pmatrix} \}\ell_2.$$

This formulation allows us to easily switch between the edges expressed in edge space and the nodes corresponding to its terminal ends.

Applicability of linear flow models

The theoretical framework in the last section has many different applications. We will demonstrate its applicability to the following systems in this section:

1. Power grids [9, 10],

2. Resistor networks [11],

3. Hydraulic networks [12, 13],

4. Limit cycle oscillators [14].

Application to power grids

The power flow equations describing the steady state of a power system at an arbitrary node i are given by [9]

$$P_i = \sum_{k=1}^{N} |V_i||V_k|(G_{ik}\cos(\vartheta_i - \vartheta_k) + B_{ik}\sin(\vartheta_i - \vartheta_k)),$$
$$Q_i = \sum_{k=1}^{N} |V_i||V_k|(G_{ik}\sin(\vartheta_i - \vartheta_k) - B_{ik}\cos(\vartheta_i - \vartheta_k)). \tag{8}$$

Here, P_i and Q_i are the real and reactive power generated or consumed at node or bus i, ϑ_i is the voltage angle at the same bus and $|V_i|$ is the voltage magnitude. The matrices $\boldsymbol{G} \in \mathbb{R}^{N\times N}$ and $\boldsymbol{B} \in \mathbb{R}^{N\times N}$ with elements G_{ij} and B_{ij}, respectively, are the real part and the complex part of the complex nodal admittance matrix $\boldsymbol{Y} = \boldsymbol{G} + i\boldsymbol{B} \in \mathbb{C}^{N\times N}$. Note that the matrices $\boldsymbol{B}$ and $\boldsymbol{G}$ are not actually matrices of susceptances and conductances, respectively. Instead, their entries read as follows

$$B_{ij} = \begin{cases} -b_{ij} & \text{if } (i,j) \in E(G),\ i \neq j, \\ b_i^{\text{shunt}} + \sum_{(i,k)\in E(G)} b_{ik} & \text{if } i = j, \\ 0 & \text{otherwise,} \end{cases}$$

where b_i^{shunt} denotes the shunt susceptance of node i and b_{ij} is the susceptance of the circuit connecting node i to node j. $\boldsymbol{G}$ has an analogous structure with elements

$$G_{ij} = \begin{cases} -g_{ij} & \text{if } (i,j) \in E(G),\ i \neq j, \\ g_i^{\text{shunt}} + \sum_{(i,k)\in E(G)} g_{ik} & \text{if } i = j, \\ 0 & \text{otherwise,} \end{cases}$$

where g_{ij} are the conductances of the circuit between nodes i and node j. The matrices $\boldsymbol{B}$ and $\boldsymbol{G}$ thus have the structure of a Laplacian matrix except for the diagonal entries which contain additional terms given by the shunt susceptances and conductances. The off-diagonal elements of the nodal admittance matrix thus read as

$$Y_{jk} = -y_{jk},\ \forall j \neq k; \quad y_{jk} = g_{jk} + ib_{jk} = \frac{1}{r_{jk} + ix_{jk}},$$

with the circuit's reactance x_{jk} and resistance r_{jk}. Note that line susceptances $b_\ell = \frac{-x}{r^2+x^2}$ are thus negative. The Eqs. (8) reduce to the lossless power flow equations in the case where the real part of the nodal admittance matrix is negligible $\boldsymbol{G} \approx \boldsymbol{0}$, i.e., lines are purely inductive.

We will focus on the so called *DC approximation* of this full AC power flow equations. This approximation is based on three assumptions [9]:

1. Voltages vary little, i.e., $|V_i| \approx \text{const},\ \forall i$ with respect to their base values,
2. Angular differences are small, i.e., $\sin(\vartheta_i - \vartheta_j) \approx \vartheta_i - \vartheta_j,\ \forall (i,j) \in E(G)$,
3. Transmission lines are purely inductive, i.e., $B_{ij} \gg G_{ij},\ \forall (i,j) \in E(G)$.

Typically, these assumptions are fulfilled for high voltage transmission grids if the line loading is not too large [15]. Using these approximations, Eq. (8) reduces to

$$P_i = \sum_{k=1}^{N} \underbrace{|V_i||V_k|B_{ik}}_{A_{ik}}(\vartheta_i - \vartheta_k),$$

thus revealing the analogy to Eq. (2).

Application to resistor networks

Resistor networks are another example which may be described using linear flow networks [16]. They have been studied for a long time leading to many fundamental results of graph theory [11]. We will briefly introduce the theory

of resistor networks and use the symbol $\hat{=}$ to refer to the corresponding quantity in the mathematical framework of linear flow networks as introduced in section 1. For resistor networks, the flow along the graph's edges is a current flow $\vec{i} \in \mathbb{R}^L \hat{=} \vec{F}$ between nodes of different voltage $\vec{V} \in \mathbb{R}^N \hat{=} \vec{\vartheta}$. The line weights are given by the inverse resistances, i.e., the conductances, of the lines $\boldsymbol{G} \in \mathbb{R}^{L\times L} \hat{=} \boldsymbol{K}$ such that Eq. (3) reads in this case

$$\vec{i} = \boldsymbol{G}\boldsymbol{I}^\top \vec{V},$$

where $\boldsymbol{I}$ is again the node-edge incidence matrix. Along the same lines, Eq. (5) translates to

$$\vec{i}_{\text{in}} = \boldsymbol{I}\vec{i}.$$

Here, $\vec{i}_{\text{in}} \in \mathbb{R}^N \hat{=} \vec{P}$ is a vector of currents injected at the graph's nodes and the Equation is again a manifestation of Kirchhoff's current law. We may thus apply the same theoretical framework to resistor networks.

Applications to hydraulic networks

The same formalism can also be shown to apply to water transport networks that we refer to as hydraulic networks or pipe networks. Consider a hydraulic network consisting of pipes that connect to each other at junctions. Then we form the underlying graph by assigning a vertex to each of the junctions and put an edge between two vertices if they are connected via a pipe. The nodal quantity of interest in this case is the pressure $\vec{p} \in \mathbb{R}^N \hat{=} \vec{\vartheta}$. If we assume the pipes to be much longer than their radius $r \ll L$ and the flow across all pipes in the network to be laminar with a Newtonian, incompressible fluid flowing through it, we can approximate the fluid flow $\vec{Q} \in \mathbb{R}^L \hat{=} \vec{F}$ across a pipe $\ell = (i, j)$ by the *Hagen–Poiseuille equation*

$$Q_\ell = K_\ell \cdot (p_i - p_j).$$

Here, we collected different parameters describing the pipe and the fluid in the line parameter

$$K_\ell = \frac{\pi r_\ell^4}{8\mu L_\ell},$$

with the pipe radius r_ℓ, the pipe length L_ℓ and the fluid's dynamic viscosity μ. Conservation of mass then requires that inflows and outflows balance as in Eq. (2). Important applications of this framework are blood vessels in humans and animals [17], the vascular system of plants [13] or hydraulic networks [18]. For vascular networks, the system does not consist of pipes but rather of smaller vascular bundles such that the scaling of line parameter K with the radius r^4 does not necessarily exactly hold [19].

Applications to limit cycle oscillators

The linear flow model may be regarded as a linearisation of the *Kuramoto model* which naturally appears in many cases, in particular when approximating weakly coupled oscillator systems near a stable limit cycle [14].

Consider a connected, simple graph $G = (E, V)$. The Kuramoto model describes a set of weakly coupled oscillators with phase angles $\vec{\vartheta} \in \mathbb{R}^N$ attached to the graph's vertices that are coupled via the graph's edges through coupling constants $A_{ij}, (i, j) \in E(G)$, see e.g. Ref. [20]. The oscillators' tendency to synchronise through the coupling is counteracted by each oscillator's natural frequency ω_j that is written compactly as a vector $\vec{\omega} = (\omega_1, ..., \omega_N)^\top \in \mathbb{R}^N$. Then the dynamics of the phase angle ϑ_i attached to node i, where $i \in \{1, ..., N\}$, reads

$$\dot{\vartheta}_i = \omega_i - \sum_k A_{ik} \sin(\vartheta_i - \vartheta_k).$$

As before, we fix an orientation of the graph's edges and summarise the coupling coefficients for all edges $(i, j) \in E(G)$ in the diagonal coupling matrix $\boldsymbol{K} \in \mathbb{R}^{L\times L}$, such that the vectorised dynamics reads

$$\dot{\vec{\vartheta}} = \vec{\omega} - \boldsymbol{I}\boldsymbol{K}\sin(\boldsymbol{I}^\top \vec{\vartheta}). \tag{9}$$

Here, $\boldsymbol{I}$ is again the graph's node-edge incidence matrix (4) and the sine function is understood to be taken element-wise, i.e

$$\sin(\boldsymbol{I}^\top \vec{\vartheta}) = (\sin([\boldsymbol{I}^\top \vec{\vartheta}]_1), ..., \sin([\boldsymbol{I}^\top \vec{\vartheta}]_L))^\top.$$

Fixed points of the dynamics are defined by a vanishing time derivative $\dot{\vec{\vartheta}} = \vec{0}$. Therefore, the equation characterising the phase angles at the fixed point $\vec{\vartheta}^*$ reads

$$\vec{\omega} = \boldsymbol{I}\boldsymbol{K}\sin(\boldsymbol{I}^\top\vec{\vartheta}^*).$$

If the angular differences on all edges are small, we may reduce this to the linear equation $\sin(\boldsymbol{I}^\top\vec{\vartheta}) \approx \boldsymbol{I}^\top\vec{\vartheta}$, again retrieving an expression analogous to the discrete Poisson equation (6).

The second-order Kuramoto model

An extension of the Kuramoto model presented in Eq. (9) is given by the second-order Kuramoto model that is also frequently used in power systems analysis to describe synchronising generators [21–23], where it is also referred to as Kuramoto model with inertia. The model contains an additional second-order time derivative of phase angles representing the generators' inertia and reads as

$$\boldsymbol{M}\ddot{\vec{\vartheta}} = -\boldsymbol{D}\dot{\vec{\vartheta}} + \vec{\omega} - \boldsymbol{I}\boldsymbol{K}\sin(\boldsymbol{I}^\top\vec{\vartheta}). \tag{10}$$

Here, $\boldsymbol{M} = \mathrm{diag}(M_1, ..., M_N) \in \mathbb{R}^{N\times N}$ and $\boldsymbol{D} = \mathrm{diag}(D_1, ..., D_N) \in \mathbb{R}^{N\times N}$ are diagonal matrices incorporating the generators' inertia coefficients and damping coefficients, respectively [21] and the other quantities are defined the same way as for the first order Kuramoto model (9). The vector of frequencies in this model corresponds to the power injections $\vec{\omega} \in \mathbb{R}^N \hat{=} \vec{P}$. Fixed points of the second order model with phase angles $\vec{\vartheta}^*$ are characterized by both, first and second order time derivative vanishing $\ddot{\vec{\vartheta}} = \dot{\vec{\vartheta}} = \vec{0}$ resulting in the same equation as for the first order model

$$\vec{\omega} = \boldsymbol{I}\boldsymbol{K}\sin(\boldsymbol{I}^\top\vec{\vartheta}^*).$$

Again, this model reduces to the linear flow model if phase differences at the fixed point are small $\sin(\boldsymbol{I}^\top\vec{\vartheta}^*) \approx \boldsymbol{I}^\top\vec{\vartheta}^*$.

Supplementary Note 2: Description of link failures

In this section, we will briefly review the analysis of link failures within the linear flow theory setting. We will first demonstrate how the effects of a link failure may be approached on the nodal level [10]. Assume that a link $k = (r, s)$ with preoutage flow $\hat{F}_k$ fails, which does not disconnect the graph. This induces a change in the potentials

$$\vec{\vartheta}' = \vec{\vartheta} + \Delta\vec{\vartheta}$$

by virtue of the discrete Poisson equation (6). Here, we introduced the vector of potential changes $\Delta\vec{\vartheta} \in \mathbb{R}^N$ and a vector of potentials after the failure $\vec{\vartheta}' \in \mathbb{R}^N$. The corresponding equation for the new grid reads as

$$\vec{P} = (\boldsymbol{L} + \Delta\boldsymbol{L})(\vec{\vartheta} + \Delta\vec{\vartheta}).$$

Here, $\Delta\boldsymbol{L}$ is the change in the Laplacian matrix due to the removal of link k and takes the form $\Delta\boldsymbol{L} = K_k\boldsymbol{I}\vec{l}_k(\boldsymbol{I}\vec{l}_k)^\top$. If we subtract the discrete Poisson equation for the old grid before the failure of link k from this equation, we arrive at the expression

$$\Delta\vec{\vartheta} = -(\boldsymbol{L} + \Delta\boldsymbol{L})^\dagger\Delta\boldsymbol{L}\vec{\vartheta}.$$

Finally, we can use the Woodbury Matrix identity to rewrite the expression into the following form [10]

$$\boldsymbol{L}\Delta\vec{\vartheta} = q_k\vec{\nu}_k, \tag{11}$$

where

$$q_k = (1 - K_k(\boldsymbol{I}\cdot\vec{l}_k)^\top\boldsymbol{L}^\dagger\boldsymbol{I}\cdot\vec{l}_k)^{-1}\hat{F}_k$$

is a source term and $\vec{\nu}_k = \vec{e}_k - \vec{e}_j$. Similar expressions appear naturally when analysing resistor networks and have been studied, for example, in Refs. [4, 24]. After calculating the potential changes based on this equation, the flow changes on a link $\ell = (\ell_1, \ell_2)$ are given by the following equation

$$\Delta F_{\ell_1\to\ell_2} = K_\ell\cdot(\Delta\vartheta_{\ell_1} - \Delta\vartheta_{\ell_2}).$$

Supplementary Note 3: Network isolators inhibit failure spreading completely

In this section we formally establish the existence of network isolators. To this end we first fix some notation.

Fundamentals and notation

We consider a linear flow network consisting of two parts, i.e. its vertex set V is written as $V = V_1 \cup V_2$. We now label the nodes in V as follows without loss of generality

$$\begin{aligned}
&1, \dots, m_1: && \text{nodes in } V_1 \text{ that are connected to } V_2 \\
&m_1 + 1, \dots, n_1: && \text{nodes in } V_1 \text{ that are not connected to } V_2 \\
&n_1 + 1, \dots, n_1 + m_2: && \text{nodes in } V_2 \text{ that are connected to } V_1 \\
&n_1 + m_2 + 1, \dots, n_1 + n_2: && \text{nodes in } V_2 \text{ that are not connected to } V_1.
\end{aligned}$$

Then the weighted adjacency matrix of the network can be written as

$$\boldsymbol{A} = \begin{pmatrix} \boldsymbol{A}_1 & \boldsymbol{A}_{12} \\ \boldsymbol{A}_{12}^\top & \boldsymbol{A}_2 \end{pmatrix},$$

$$\boldsymbol{A}_{12} = \begin{pmatrix} \boldsymbol{a} & \boldsymbol{0} \\ \boldsymbol{0} & \boldsymbol{0} \end{pmatrix},$$

with $\boldsymbol{A}_1 \in \mathbb{R}^{n_1 \times n_1}$, $\boldsymbol{A}_2 \in \mathbb{R}^{n_2 \times n_2}$, $\boldsymbol{A}_{12} \in \mathbb{R}^{n_1 \times n_2}$ and $\boldsymbol{a} \in \mathbb{R}^{m_1 \times m_2}$. Furthermore, we define the degree matrices $\boldsymbol{D}_1$, $\boldsymbol{D}_2$ and $\boldsymbol{d}$ associated with the adjacency matrices $\boldsymbol{A}_1$, $\boldsymbol{A}_2$ and $\boldsymbol{a}$, that is

$$d_{kl} = \begin{cases} \sum_p a_{kp} & \text{for } \; k = l \\ 0 & \phantom{\text{for }} \; k \neq l \end{cases},$$

and the Laplacian matrices $\boldsymbol{L}_1 = \boldsymbol{D}_1 - \boldsymbol{A}_1$ of subnetwork 1, $\boldsymbol{L}_2 = \boldsymbol{D}_2 - \boldsymbol{A}_2$ of subnetwork 2 and $\boldsymbol{L}$ of the whole system.

Main theorem on network isolators

In this subsection, we proof the main Theorem 1 on network isolators. Consider the Theorem on network isolators.

Theorem 1. Consider a linear flow network composed of two modules 1,2 and let $\mathbf{A}_{12}$ denote the weighted adjacency matrix of the mutual connections. An edge failure in one module does not affect the flows in the other module if $\text{rank}(\mathbf{A}_{12}) = 1$. For unweighted networks this criterion is fulfilled if $\mathbf{A}_{12}$ describes a complete bipartite graph.

Proof. Assume that the adjacency matrix of the mutual connections has unit rank $\text{rank}(\boldsymbol{A}_{12}) = \text{rank}(\boldsymbol{a}) = 1$. We first proof that for any vector $\vec{y} \in \mathbb{R}^{n_1}$ the following statement holds

$$\vec{x} = \begin{pmatrix} \boldsymbol{d}^{-1}\boldsymbol{a} & \boldsymbol{0} \\ \boldsymbol{0} & \boldsymbol{0} \end{pmatrix} \vec{y} = c \begin{pmatrix} 1 \\ \vdots \\ 1 \\ 0 \\ \vdots \\ 0 \end{pmatrix}, \tag{12}$$

where $c \in \mathbb{R}$ is some real number. This result can be obtained by writing $\vec{x} \in \mathbb{R}^{n_2}$ in components. For all $j \in \{1, \dots, m_2\}$ we have

$$x_j = \frac{\sum_k a_{jk} y_k}{\sum_k a_{jk}}.$$

Since $\boldsymbol{a}$ has unit rank all its rows are linearly dependent such that we can write $a_{jk}/a_{1k} = a_{j1}/a_{11}$ for all $k \in \{1, \ldots, n_1\}$, such that $a_{jk} = a_{1k}a_{j1}/a_{11}$. Hence,

$$\begin{aligned} x_j &= \frac{a_{j1}/a_{11} \times \sum_k a_{1k} y_k}{a_{j1}/a_{11} \times \sum_k a_{1k}} \\ &= \frac{\sum_k a_{1k} y_k}{\sum_k a_{1k}} = x_1 =: c, \end{aligned}$$

and all elements of the vector are equal. The remaining $n_2 - m_2$ elements of the vector vanish, $x_j = 0$, $\forall j \in \{m_2 + 1, ..., n_2\}$, because the corresponding adjacency matrix $\boldsymbol{A}_{12}$ has only zero entries at the respective positions.

We now compute the impact of a failure of link k in $G(V_1)$ via the discrete Poisson equation (11)

$$\boldsymbol{L}\Delta\vec{\vartheta} = q_k \vec{\nu}_k.$$

We decompose this equation as well as the vectors $\Delta\vec{\vartheta}$ and $\vec{\nu}$ into two parts corresponding to the two parts of the network

$$\Delta\vec{\vartheta} = \begin{pmatrix} \Delta\vec{\vartheta}_1 \\ \Delta\vec{\vartheta}_2 \end{pmatrix}, \qquad \vec{\nu} = \begin{pmatrix} \vec{\nu}_1 \\ \vec{0} \end{pmatrix},$$

where $\Delta\vec{\vartheta}_1, \vec{\nu}_1 \in \mathbb{R}^{n_1}$ and $\Delta\vec{\vartheta}_2, \vec{\nu}_2 \in \mathbb{R}^{n_2}$. Then the lower part of Eq. (11) corresponding to the vertices $n_1 + 1, \ldots, n_1 + n_2$ reads

$$\left[\boldsymbol{L}_2 + \begin{pmatrix} \boldsymbol{d} & \boldsymbol{0} \\ \boldsymbol{0} & \boldsymbol{0} \end{pmatrix}\right] \Delta\vec{\vartheta}_2 = \begin{pmatrix} \boldsymbol{a} & \boldsymbol{0} \\ \boldsymbol{0} & \boldsymbol{0} \end{pmatrix} \Delta\vec{\vartheta}_1, \tag{13}$$

using the notation established above. Using the prior result (12) and multiplying by the matrix

$$\begin{pmatrix} \boldsymbol{d}^{-1} & \boldsymbol{0} \\ \boldsymbol{0} & \boldsymbol{1} \end{pmatrix},$$

this equation can be rewritten as

$$\left[\begin{pmatrix} \boldsymbol{d}^{-1} & \boldsymbol{0} \\ \boldsymbol{0} & \boldsymbol{1} \end{pmatrix} \boldsymbol{L}_2 + \begin{pmatrix} \boldsymbol{1} & \boldsymbol{0} \\ \boldsymbol{0} & \boldsymbol{0} \end{pmatrix}\right] \Delta\vec{\vartheta}_2 = \begin{pmatrix} \boldsymbol{d}^{-1}\boldsymbol{a} & \boldsymbol{0} \\ \boldsymbol{0} & \boldsymbol{0} \end{pmatrix} \Delta\vec{\vartheta}_1 = c \begin{pmatrix} 1 \\ \vdots \\ 1 \\ 0 \\ \vdots \\ 0 \end{pmatrix}.$$

Now one can easily check via a direct calculation that

$$\Delta\vec{\vartheta}_2 = c \begin{pmatrix} 1 \\ \vdots \\ 1 \end{pmatrix}$$

is a solution to this equation. Furthermore, this solution is unique as the linear system of equation has full rank. This is most easily seen for Eq. (13), as the matrix on the left hand side is normal and positive definite.

We have thus shown that the nodal potentials in V_2 are shifted by the same constant c when a link in $G(V_1)$ fails. Hence the flow changes are given by

$$\Delta F_{\ell_1 \to \ell_2} = A_\ell(\Delta\vartheta_{\ell_1} - \Delta\vartheta_{\ell_2}) = 0 \quad \forall \ell_1, \ell_2 \in V_2.$$

□

Corollary 1 (Complete bipartite graphs are network isolators). Consider a linear flow network consisting of two modules with vertex sets V_1 and V_2 and assume that a single link in the induced subgraph $G(V_1)$ fails, i.e. a link (r, s) with $r, s \in V_1$. If the subgraph G' of mutual connections between the two modules is a complete bipartite graph with uniform edge weights $K = K_\ell = K_m$, $\forall \ell, m \in E(G')$, then the subgraph is a network isolator. If the whole graph is unweighted, G' always has uniform edge weights, thus a complete bipartite graph of mutual connections always is a network isolator for any unweighted network.

Proof. If the subgraph G' is complete and bipartite (ignoring all connections within both induced subgraphs $G(V_1)$ and $G(V_2)$), its adjacency matrix takes the form

$$\boldsymbol{A}' = K \cdot \begin{pmatrix} \mathbf{0} & \mathbf{1}_{m_1 \times m_2} \\ \mathbf{1}^\top_{m_1 \times m_2} & \mathbf{0} \end{pmatrix}.$$

We can immediately see that the matrix in the upper right corner, i.e. $\boldsymbol{A}'_{12} = K\mathbf{1}_{m_1 \times m_2}$ has unit rank, such that by theorem 1, G' is a network isolator. □

Network isolators in non-linear systems

We will now demonstrate how to extend the concepts of network isolators from linear systems to a certain class of non-linear networked systems

$$\vec{f}(\boldsymbol{L}\vec{x}) = (f_1([\boldsymbol{L}\vec{x}]_1), ..., f_N([\boldsymbol{L}\vec{x}]_N))^\top : \vec{x} \in \mathbb{R}^N \to \vec{f}(\boldsymbol{L}\vec{x}) \in \mathbb{R}^N$$

be a continuous function on the real numbers that depends on the product of Laplacian matrix $\boldsymbol{L}$ and vector $\vec{x}$. Here, $[\boldsymbol{L}\vec{x}]_j$ denotes the j-th row of the standard matrix-vector product $\boldsymbol{L}\vec{x}$. We assume that the underlying network topology is again separated into two subgraphs $G(V_1)$ and $G(V_2)$, see the beginning of this section. We further assume that

$$f_j(0) = 0, \; \forall j \in \{1, ...N\},$$

i.e., each of the functions vanishes at the origin. Note that the functions $f_j([\boldsymbol{L}\vec{x}]_j)$ can be different and non-linear, as long as they vanish at the origin. Consider a dynamical system of the form

$$\dot{\vec{x}} = \vec{f}(\boldsymbol{L}\vec{x}) \tag{14}$$

that admits a fixed point solution $\vec{x}^*$ with vanishing time derivative $\dot{\vec{x}} = \vec{0}$ that fulfils

$$\vec{0} = \vec{f}(\boldsymbol{L}\vec{x}^*). \tag{15}$$

Now add a perturbation vector

$$\Delta\vec{P} = \begin{pmatrix} \Delta\vec{P}_1 \\ \vec{0} \end{pmatrix} \tag{16}$$

to the system that has non-zero entries only at the nodes of the first induced subgraph $G(V_1)$ and assume that the dynamical system (14) relaxes to a new fixed point $\vec{x}'$ with

$$\Delta\vec{P} = \vec{f}(\boldsymbol{L}\vec{x}'). \tag{17}$$

Then the following corollary holds

Corollary 2 (Isolation in non-linear systems). Consider a non-linear dynamical networked system of the form (14) that consists of two modules with vertex sets V_1 and V_2 which are connected by a network isolator as of Theorem 1. Assume that the system admits a fixed point solution as given in Eq. (15). Assume that a perturbation as in Eq. (16) is applied to the nodes in the first induced subgraph $G(V_1)$ and that the system relaxes to a new fixed point as in Eq. (17). Then the new fixed point has the following form

$$\vec{x}' = \begin{pmatrix} \vec{x}'_1 \\ c\vec{1}_2 \end{pmatrix},$$

where $c \in \mathbb{R}$ is a constant.

The second module is thus isolated against perturbations in the first module and vice versa in the sense that a perturbation in one module results in a constant shift in the other module.

Proof. The proof is analogous to the proof of Theorem 1. Applying the function $\vec{f}$ to Eq. (13) describing the fixed point in the non-perturbed subgraph $G(V_2)$, we see that the system is still solved by

$$\vec{x}' = \begin{pmatrix} \Delta\vec{x}'_1 \\ c\vec{1}_2 \end{pmatrix}.$$

□

Even if not rigorously valid, we find that strong network isolation persists for an even larger class of non-linear systems that we will discuss in this section. Note that our analysis here closely follows a linear response theory analysis of Kuramoto oscillators that can be found in Ref. [14].
Consider a networked non-linear dynamical system of the form

$$\dot{\vec{x}}_i = \vec{f}(\vec{x}_i)_i + \sum_{k=1}^{N} A_{ik} g(x_i - x_k). \tag{18}$$

Here, $\vec{x} \in \mathbb{R}^N$ is a vector of nodal dynamical variables, $\vec{f}$ is a differentiable function of self-interactions of these variables and $\vec{g}(\vec{x})$ is a differentiable, odd function that depends only on the differences of nodal variables at neighbouring nodes. Odd functions are characterised by the property that $\vec{g}(-\vec{x}) = -\vec{g}(\vec{x})$ and this property results in a diffusive coupling between neighbouring nodes as is present for example in case of the sinusoidal coupling used in the Kuramoto model (see Eq. (9)). The strength of interactions is encoded in the graph's adjacency matrix $\boldsymbol{A}$. Assume that the system relaxes to a fixed point with $\dot{\vec{x}}_i = 0$ where $\vec{x}(t) = \vec{x}^*$. If we perturb the network locally at a node or an edge, we can compute the change in this fixed point using linear response theory [14]: to leading order, we obtain a linear system as above.
Assume that we perturb a single edge (n, m) by modifying its edge weight by a small number ΔA_{ij} such that

$$A_{ij} \to A'_{ij} = A_{ij} + \Delta A_{ij}$$
$$\Delta A_{ij} = \begin{cases} 0 & \text{if } (i,j) \neq (n,m) \\ \Delta A & \text{if } (i,j) = (n,m) \end{cases} .$$

Assume that this modification causes a change of the fixed point by

$$x_j^* \to x_j' = x_j^* + \Delta x_j, \quad \forall j \in \{1, ..., N\},$$

where Δx_j is the change in the fixed point that is assumed to be small such that the fixed points lie closed to each other. We can expand the dynamics to leading order in terms of the new fixed point

$$\frac{\partial f(x_j^*)}{\partial x_j} \Delta x_j + \sum_{k=1}^{N} A_{jk} \frac{\partial g(x_j^* - x_k^*)}{\partial x_j} (\Delta x_j - \Delta x_k) + s_j = 0.$$

Here, s_j is a source term that vanishes if node j is not part of the edge (n, m), $j \neq n, m$. The sum in this expression may be compactly written in terms of an effective Laplacian matrix $\tilde{\boldsymbol{L}}$

$$\sum_{k=1}^{N} A_{jk} \frac{\partial g(x_j^* - x_k^*)}{\partial x_j} (\Delta x_j - \Delta x_k) = [\tilde{\boldsymbol{L}} \Delta \vec{x}]_j,$$

where the Laplacian matrix has the off-diagonal entries

$$\tilde{\boldsymbol{L}}_{jk} = -A_{jk} \frac{\partial g(x_j^* - x_k^*)}{\partial x_j}.$$

Thus, if the underlying graph contains a network isolator, we can apply Theorem 1 to the system and see immediately that each component is (approximately) isolated against small perturbations in the other one. Note that this result is only valid if the change in the fixed point as well as the perturbation are small and relies on the fact that the system relaxes to a new fixed point after the perturbation. In particular, this description applies to Kuramoto oscillators (Eq. (9)) perturbed at a few nodes or edges and powergrids described by AC load flow equations 8 subject to a link failure. We can thus get approximate isolation in both models as shown in Figure 5 for the AC load flow model and Figures 4 and Supplementary Figure 10 for the Kuramoto model.

Supplementary Note 4: Linear controllability of complex networks

We now turn to a different theoretical concept in complex networks research: the controllability of a network. In this section, we briefly analyse the influence of network isolators on the controllabilty of complex systems with a linear

dynamics. In general, we find that introducing a network isolator to a complex network has no generic influence on its controllability.
Consider a linear dynamical system on a network with N nodes with a state vector $\vec{x} \in \mathbb{R}^N$ whose dynamics is given by [2]

$$\dot{\vec{x}} = \boldsymbol{A}\vec{x} + \boldsymbol{B}\vec{u}. \tag{19}$$

Here, $\boldsymbol{A} \in \mathbb{R}^{N\times N}$ denotes the graph's adjacency matrix, $\vec{u} \in \mathbb{R}^m$ is a (potentially time-varying) input vector that is supposed to achieve control of the network and $\boldsymbol{B} \in \mathbb{R}^{N\times m}$ is the control matrix. Then one definition of controllability is the following: Can we find a set of m driver nodes identified by the controllability matrix $\boldsymbol{B}$ such that the system may be driven from any initial state $\vec{x}_0$ to any final state $\vec{x}_f$ in finite time? If yes, the system is said to be *controllable* and a measure of its controllability is given by the minimum number of driving nodes $N_d \leq N$ necessary to achieve full controllability [2, 25, 26].
We identify this set of driver nodes necessary for exact controllability for a small sample network using a method due to Yuan et al. [2] who demonstrated that the minimum number of driver nodes N_d can be found by determining the multiplicity of the eigenvalues of the graph's adjacency matrix $\mathbf{A}$ [2]. Assume that the underlying network is undirected such that its adjacency matrix is symmetric as for the networks studied in this manuscript. In this case, we can calculate the algebraic multiplicity $\delta(\lambda_i)$ for all eigenvalues λ_i of this matrix to calculate the minimum number of driver nodes, N_D, necessary to control the network (cf. Eq.4, Ref. [2])

$$N_D = \max_i \left[\delta(\lambda_i)\right]. \tag{20}$$

This approach has the advantage that the driver nodes necessary to control the network, i.e., the controllability of a network, may immediately be identified, which is more complicated when using the classical Kalman rank condition [2].
In Figure 8, we illustrate a potential application of this formalism to network isolators. The adjacency matrix of the graph reported in panel (a) has the eigenvalue $\lambda_M = -1$ with multiplicity $\delta(\lambda^M) = 2$, while all other eigenvalues have multiplicity one. An eigenvalue $\lambda_M = -1$ in the adjacency matrix can easily be constructed by connecting two nodes to the other nodes in a network in exactly the same way [4]. Thus, by the criterion (20), only two nodes are required to control the network. These nodes have been determined using the method described in Ref. [2] and are highlighted in orange. After introducing the isolator into the system (panel (d)), the maximum multiplicity of any eigenvalue of the graph's adjacency matrix is one, i.e., $\delta(\lambda_i) = 1,\ \forall i$, which implies that the graph can be controlled by a single node (colored red). Therefore, in this case, the controllability of the network is increased after constructing the isolator. We emphasize that the network isolator prevents only flow changes, but not flows from passing as demonstrated in panels (b,c) and (e,f) .
For the remaining network isolators constructed in throughout this manuscript, we did not find any influence of the introduction of network isolators on the controllability of the underlying network and thus conclude that isolators do not generically influence network controllability.

SUPPLEMENTARY REFERENCES

[1] Hörsch, J., Hofmann, F., Schlachtberger, D. & Brown, T. PyPSA-eur: An open optimisation model of the european transmission system. *Energy Strategy Reviews* **22**, 207–215 (2018).
[2] Yuan, Z., Zhao, C., Di, Z., Wang, W.-X. & Lai, Y.-C. Exact controllability of complex networks. *Nature Communications* **4**, 2447 (2013).
[3] Newman, M. E. J. *Networks: An introduction* (Oxford University Press, 2010).
[4] Van Mieghem, P., Devriendt, K. & Cetinay, H. Pseudoinverse of the laplacian and best spreader node in a network. *Physical Review E* **96**, 032311 (2017).
[5] Dörfler, F., Simpson-Porco, J. W. & Bullo, F. Electrical networks and algebraic graph theory: Models, properties, and applications. *Proceedings of the IEEE* **106**, 977–1005 (2018).
[6] Radicchi, F., Castellano, C., Cecconi, F., Loreto, V. & Parisi, D. Defining and identifying communities in networks. *Proceedings of the National Academy of Sciences* **101**, 2658–2663 (2004).
[7] Girvan, M. & Newman, M. E. J. Community structure in social and biological networks. *Proceedings of the National Academy of Sciences* **99**, 7821–7826 (2002).
[8] Newman, M. E. J. Communities, modules and large-scale structure in networks. *Nature Physics* **8**, 25–31 (2012).
[9] Wood, A. J., Wollenberg, B. F. & Sheblé, G. B. *Power Generation, Operation and Control* (John Wiley & Sons, New York, 2014).
[10] Strake, J., Kaiser, F., Basiri, F., Ronellenfitsch, H. & Witthaut, D. Non-local impact of link failures in linear flow networks. *New Journal of Physics* **21**, 053009 (2019).
[11] Bollobás, B. *Modern graph theory.* No. 184 in Graduate texts in mathematics (Springer, 1998).

[12] Sack, L., Dietrich, E. M., Streeter, C. M., Sanchez-Gomez, D. & Holbrook, N. M. Leaf palmate venation and vascular redundancy confer tolerance of hydraulic disruption. *Proceedings of the National Academy of Sciences* **105**, 1567–1572 (2008).
[13] Katifori, E., Szöllősi, G. J. & Magnasco, M. O. Damage and fluctuations induce loops in optimal transport networks. *Physical Review Letters* **104**, 048704 (2010).
[14] Manik, D. *et al.* Network susceptibilities: Theory and applications. *Physical Review E* **95**, 012319 (2017).
[15] Purchala, K., Meeus, L., Dommelen, D. V. & Belmans, R. Usefulness of dc power flow for active power flow analysis. In *IEEE Power Engineering Society General Meeting*, 454–459 Vol. 1 (2005).
[16] Kaiser, F. & Witthaut, D. Topological theory of resilience and failure spreading in flow networks. *arXiv:2009.10349 [physics]* (2020). URL `http://arxiv.org/abs/2009.10349`. ArXiv: 2009.10349.
[17] Reichold, J. *et al.* Vascular graph model to simulate the cerebral blood flow in realistic vascular networks. *Journal of Cerebral Blood Flow & Metabolism* **29**, 1429–1443 (2009).
[18] Wurbs, R. A. & James, W. P. *Water Resources Engineering* (Pearson, 2001).
[19] Coomes, D. A., Heathcote, S., Godfrey, E. R., Shepherd, J. J. & Sack, L. Scaling of xylem vessels and veins within the leaves of oak species. *Biology Letters* **4**, 302–306 (2008).
[20] Rodrigues, F. A., Peron, T. K. D., Ji, P. & Kurths, J. The kuramoto model in complex networks. *Physics Reports* **610**, 1–98 (2016).
[21] Rohden, M., Sorge, A., Timme, M. & Witthaut, D. Self-organized synchronization in decentralized power grids. *Phys. Rev. Lett.* **109**, 064101 (2012).
[22] Nishikawa, T. & Motter, A. E. Comparative analysis of existing models for power-grid synchronization. *New Journal of Physics* **17**, 015012 (2015).
[23] Manik, D. *et al.* Supply networks: Instabilities without overload. *The European Physical Journal Special Topics* **223**, 2527–2547 (2014).
[24] Biggs, N. Algebraic potential theory on graphs. *Bulletin of the London Mathematical Society* **29**, 641–682 (1997).
[25] Gao, J., Liu, Y.-Y., D'Souza, R. M. & Barabási, A.-L. Target control of complex networks. *Nature Communications* **5**, 5415 (2014).
[26] Liu, Y.-Y., Slotine, J.-J. & Barabási, A.-L. Controllability of complex networks. *Nature* **473**, 167–173 (2011).
